# Using nanopore metagenomics to characterize pathogens in febrile patients from a highland of Western Kenya

**DOI:** 10.3389/fcimb.2026.1884846

**Published:** 2026-08-12

**Authors:** Felix M. Pabon-Rodriguez, George Ayodo

**Affiliations:** 1 Department of Biostatistics and Health Data Science, Indiana University School of Medicine, Indianapolis, IN, United States; 2 Centre for Community Health and Wellbeing, Jaramogi Oginga University of Science and Technology, Bondo, Kenya

**Keywords:** febrile illnesses, malaria infection, nanopore metagenomics, surveillance, Western Kenya

## Abstract

**Introduction:**

Febrile illness remains a leading cause of morbidity in sub-Saharan Africa despite substantial reductions in malaria transmission. As malaria positive cases decrease, an increasing number of patients with febrile illnesses test negative for *Plasmodium* infection, leaving their causes unresolved. As a result of the diagnostic gap, patients receive presumptive antimalarial treatment, unnecessary antibiotics and experience delayed therapy.

**Methods:**

We conducted a nanopore metagenomic next-generation sequencing (mNGS) investigation of 168 archived blood samples collected from febrile patients in Kipsamoite and Kapsisywa sites in Nandi County, a highland region in western Kenya experiencing declining malaria transmission, between 2012 and 2020. The study used exploratory taxonomic profiling to identify microbial DNA/RNA signatures in archived plasma samples and described organisms with known or potential clinical relevance, while classifying detected organisms into common commensal, skin-associated, and environmental taxa. Demographic and clinical data linked to samples were used to fit multivariable logistic regression models to assess associations.

**Results:**

Sequencing revealed substantial microbial heterogeneity, including frequent detection of common skin/environmental taxa, opportunistic organisms, ubiquitous viruses, and a smaller number of organisms with established fever-causing potential. The detections are interpreted as molecular evidence of microbial nucleic acid, not as proof of active infection or fever causality. Malaria-positive subjects had a higher mean number of identified pathogens compared to malaria-negative subjects (3.78 vs. 2.14; p = 0.002), despite a baseline microbial landscape dominated by commensal flora, environmental organisms, and ubiquitous viruses.

**Discussion:**

Nanopore mNGS is a viable tool for identifying non-malarial febrile pathogens in western Kenya. Future efforts must combine systematic sampling with field-deployable contamination controls and causal confirmation frameworks to optimize regional antimicrobial stewardship and surveillance.

## 1 Introduction

Fever without a localized cause is one of the most common reasons for healthcare utilization in sub-Saharan Africa, including Western Kenya (Prasad et al., 2015a). However, many cases are reported in areas of high malaria transmission (O’Meara et al., 2015; Maze et al., 2018; Elven et al., 2020; Verani et al., 2024). In areas of low, unstable malaria transmission, fever (axillary temperature ≥37.5 °C) is a sensitive indicator of clinical malaria (Mutanda et al., 2014), but fever is also caused by a diversity of pathogens across geographic settings, seasons and populations (Baltzell et al., 2019; Halliday et al., 2020). Consequently, acute, sub-acute and chronic fevers based on duration have been linked to different disease-causing agents (Surana et al., 2022). The sustained malaria control efforts have resulted in substantial declines in malaria transmission (Hamre et al., 2020a), leading to a growing proportion of patients presenting with fever but testing negative for *Plasmodium* species (Ogoina, 2011). This shift has created a considerable burden of non-malarial febrile illness (NMFI), a category of illness whose etiologies remain insufficiently characterized in many sub-Saharan Africa (SSA) settings due to diagnostic limitations (Mutanda et al., 2014; Crump and Kirk, 2015; Prasad et al., 2015b). Across SSA, multiple studies suggest that the majority of febrile illnesses have non-malarial origins, with bacterial pathogens accounting for roughly half of detected etiologies and viral agents comprising another third (Prasad et al., 2015a; Prasad et al., 2015b; Elven et al., 2020; Wainaina et al., 2022). Surveillance studies similarly show that significant proportions of febrile cases remain undiagnosed despite targeted molecular testing, highlighting persistent diagnostic blind spots (Dalrymple et al., 2019; Kairu-Wanyoike et al., 2019; Roddy et al., 2019; Shrestha et al., 2020; Levine et al., 2024; Mandai et al., 2024; Sabbaghian et al., 2024; Verani et al., 2024; Ashraf et al., 2025; Belarbi et al., 2025; Gao et al., 2025; Postigo-Hidalgo et al., 2025). These gaps reflect the constraints of conventional diagnostic tools, which are often limited to malaria rapid diagnostic tests and basic cultures, with little capacity for detecting fastidious, anaerobic, viral, zoonotic, or emerging pathogens. As a result, clinical management frequently defaults to empiric antimicrobial therapy, contributing to inappropriate antibiotic use (Maze et al., 2018; Pokharel et al., 2024; Zhang et al., 2024).

Metagenomic next-generation sequencing (mNGS) has emerged as a powerful, hypothesis-free tool capable of detecting a broad spectrum of microbial taxa (bacteria, viruses, fungi, and parasites) from a single sample. Recent work has demonstrated the feasibility of using nanopore-based mNGS in resource-limited contexts, where its portability, rapid turnaround, and long-read capabilities allow for on-site or near-site pathogen detection (Faria et al., 2016; Quick et al., 2016; Kafetzopoulou et al., 2019; Di Paola et al., 2020). Implementation of mNGS in Uganda and Kenya have identified previously undetected pathogens in patients with acute febrile and undifferentiated illnesses (sudden onset of fever, typically ≥38 °C, lasting less than two weeks that lacks localized organ symptoms or a clear source after an initial clinical evaluation), including Rickettsia, hepatitis A virus, Rift Valley fever virus, and other agents missed by conventional diagnostics (Kairu-Wanyoike et al., 2019; Sabbaghian et al., 2024). Additionally, *anelloviruses* are common components of the human blood virome and are frequently detected in both healthy and ill individuals (Sabbaghian et al., 2024).

Despite these advances, a few studies have systematically applied nanopore-based mNGS to archived plasma specimens from febrile patients in SSA. Addressing this gap, the present study leverages a well-characterized, longitudinally collected archive of blood samples from febrile patients in Western Kenya (2012–2020) with the goal of (1) describe microbial nucleic-acid signatures identified by nanopore mNGS in archived plasma specimens from febrile patients; (2) quantify pathogen prevalence; (3) differentiate commensal and opportunistic organisms from established febrile pathogens; and (4) generate potential explanation regarding NMFI etiologies in a setting where malaria decline has reshaped clinical epidemiology. This work has implications for diagnostics, surveillance, and antimicrobial stewardship in resource-limited settings.

## 2 Methods

### 2.1 Study population and sample repository

Archived blood samples were obtained between 2012 and 2020 from febrile hospitalized patients (axillary temperature ≥37.5 °C) presenting to Kipsamoite and Kapsisywa outpatient healthcare facilities in Nandi County a highland in Western Kenya. Elevation ranges from 1,887 to 2,132 meters (m) above sea level. The area experiences highly seasonal malaria transmission, with a primary peak from May to July following the long rains (March-June). While short rains (October-November) can trigger a secondary spike, dry intervals suppress vector breeding, keeping baseline transmission low but vulnerable to outbreaks (Githinji et al., 2024). In addition, over a twenty-year period, the combined facility catchments expanded from approximately 6,400 to over 9,000 residents (John et al., 2009; Hamre et al., 2020b). From the parent study, to collect plasma, the study team drew 3 to 5 mL of venous blood from children and 10 to 15 mL from adults into heparinized vacutainer tubes and processed the samples within six hours of collection. The plasma was separated using density gradient centrifugation and subsequently frozen and stored at −20 °C (or lower) for long-term analyses (Ayieko et al., 2013). Samples were linked to basic demographic and clinical metadata from the parent study database, including age, sex, date of collection, fever status, and malaria test results. Additional information included village identification, proximity indicator to river/forest, latitude, longitude, and elevation of the household. Age was categorized as 0-5, 6-15, and >=16 years. These categories were selected *a priori* to reflect broad epidemiologic strata relevant to malaria and febrile illness surveillance: young children, school-aged children/adolescents, and adults. Children under 5 years are commonly treated as a distinct high-risk pediatric group in malaria and febrile illness studies, while school-aged children (6–15 years) are increasingly recognized as an important group for malaria infection burden and transmission (Walldorf et al., 2015; Cohee et al., 2021; Okongo et al., 2025). Using fever and malaria status, two primary groups were defined: (1) patients with malarial fever and (2) patients with non-malarial fever. A total of 1,396 plasma samples were available after conclusion of the parent study, including 95 individuals with malarial fever and 1,301 individuals with non-malarial fever. To preserve the privacy of the samples, GPS coordinates and names of the villages are not provided throughout the manuscript nor explicitly used in any tables or graphs. These samples were collected following the parent study protocol, which allowed future pathogen-related testing.

Due to plasma volume requirements and the analytical advantages of unbiased pathogen detection, metagenomic next-generation sequencing (mNGS) was chosen as the diagnostic method for the study. Only samples with ≥200 µL of plasma were eligible for mNGS, resulting in the inclusion of 23 samples from individuals with malarial fever and 145 samples from individuals with non-malarial fever, for a total of N = 168 individuals. Since the mNGS subset was selected on the basis of available archived plasma volume, this should not be interpreted as a random sample of the full repository. Because selection was constrained by residual sample availability, the possibility of selection bias related to collection year, storage history, participant characteristics, malaria status, or disease severity cannot be excluded. Although additional samples were available with sufficient volume for amplicon sequencing (16S rRNA/ITS), these were not included in the present analysis. The remaining archived samples will be processed in the future when additional funding becomes available, allowing expansion of metagenomic analyses and complementary amplicon-based profiling.

### 2.2 Metagenomic sequencing and bioinformatics

DNA (deoxyribonucleic acid) was extracted from plasma samples and prepared for nanopore sequencing following established laboratory protocols. Metagenomic sequencing was performed to identify bacterial, fungal, and viral pathogens in plasma samples using the Rapid PCR Barcoding Kit V14 (SQK-RPB114.24) and the RMS_9215_v114_revF_18Nov2025 protocol from Oxford Nanopore Technologies. For each patient, 200 μL of plasma was centrifuged at 10,000 × g for 5 minutes. The supernatant was transferred to a fresh tube for viral nucleic acid extraction, and the pellet was reserved for bacterial and fungal DNA isolation. Host nucleic acid depletion was performed using HL-SAN endonuclease (ArcticZymes Technologies) prior to viral nucleic acid extraction with the MagMAX Viral/Pathogen Ultra Nucleic Acid Isolation Kit (Applied Biosystems). Reverse transcription was carried out on the purified viral nucleic acid using a 9N primer, a template-switching oligonucleotide, and Maxima H Minus Reverse Transcriptase (Thermo Scientific™). The reverse transcribed viral nucleic acid was amplified by PCR using Rapid Barcode Primers (Oxford Nanopore). Bacterial and fungal DNA was isolated from the plasma pellet following saponin-based washing and HL-SAN treatment, using the same MagMAX extraction kit. Tagmentation was performed to fragment the extracted bacterial and fungal DNA and following the rapid barcode PCR (Oxford Nanopore). The barcoded viral DNA and microbial DNA was pooled in one tube and treated with Exonuclease I (NEB) cleanup, then purified with AMPure XP (Oxford Nanopore). The barcoded DNA Libraries were sequenced on a MinION platform using R10.4.1 flow cells (FLO-MIN114, Oxford Nanopore), with basecalling performed in MinKNOW (version 25.09.16). Taxonomic classification and metagenomic analysis were conducted using the EPI2ME with wf-metagenomic workflow, employing minimap2 (v2.28-r1209) for read alignment, samtools (v1.21) for alignment processing, Kraken (v2.1.3) for k-mer-based taxonomic classification, taxonkit (v0.19.0) for taxonomy manipulation, fastcat (v0.20.0) for read concatenation and quality filtering, and pysam (v0.23.0) and pandas (v2.2.3) for downstream data handling and analysis.

Batch-matched negative extraction controls and no-template controls were not available for all sequencing batches; therefore, formal background subtraction against matched controls was not performed. To reduce overinterpretation, organisms commonly associated with skin, oral, reagent, or environmental sources were classified *a priori* as common microbiome/environmental taxa or possible contaminants, and these organisms were not interpreted as definitive causes of fever. Taxonomic calls were generated using EPI2ME wf-metagenomics with Kraken2 classification and the built-in Standard-8 database set. Organisms were retained for descriptive analysis when the workflow produced genus- or species-level taxonomic assignments; results were summarized at the individual level as presence/absence rather than as quantitative organism burden. Because batch-specific negative controls were unavailable, all low-abundance detections and taxa typical of skin, reagent, or environmental contamination were interpreted cautiously.

Complete sequencing protocol and additional details about EPI2ME reports, including workflow parameters and QC values, are provided in the **Supplementary Material**.

### 2.3 Statistical analysis

Pathogen prevalence was summarized across the cohort included in the metagenomic. Results are presented as counts and proportions, reflecting organism detection at the individual level. Each individual was counted once per organism (presence/absence), regardless of read depth. Microorganisms were categorized before interpretation into three non-mutually causal interpretation groups: (1) common microbiome, skin-associated, oral, reagent-associated, or environmental taxa; (2) opportunistic organisms with documented pathogenic potential under appropriate host or exposure conditions; and (3) organisms with established fever-causing or invasive disease potential in the literature. Categorization was based on organism-level clinical plausibility, known ecological niche, prior reports of bloodstream or systemic infection, and likelihood of contamination in plasma metagenomic sequencing. These categories were used to guide interpretation and not to establish causality. The operational criteria for these pathogen categories are included in the **Supplementary Material**. Prevalence was calculated as the number of individuals in whom a given organism was detected.

Demographic and clinical characteristics were summarized by malaria status (positive vs. negative). Continuous variables are reported as mean, median, and standard deviation; categorical variables are reported as counts and percentages. Between-group comparisons were performed using chi-squared tests or Kruskal-Wallis tests. Spatial characteristics, including household elevation and distance to the nearest health clinic, were derived from linked GIS data and included in descriptive summaries. The temporal distribution of pathogen types was examined by calendar month, stratified by malaria status, and visualized as stacked bar charts. Spatial distribution of cases was mapped using household GPS coordinates, with villages delineated by convex hull boundaries. The number of identified pathogens was summarized by village using boxplots with individual data points overlaid.

To examine the association between clinical and demographic factors and non-malarial fever, a multivariable logistic regression model was fit with non-malarial fever (vs. malarial fever) as the binary outcome. Covariates included calendar month, sex, age, distance to clinic, and household elevation. Month was modeled as an ordinal variable (1–12) reflecting calendar month. We recognize that this specification does not fully capture cyclic seasonality; therefore, results are interpreted as exploratory evidence of temporal patterning rather than as a definitive seasonal model. Results are reported as odds ratios (ORs) with 95% confidence intervals. Univariable and multivariable estimates are presented in a merged regression table. A likelihood ratio test was used to assess whether calendar month contributed significantly to model fit beyond the null model. To assess temporal trends in pathogen burden, a zero-inflated Poisson regression model was fit with the number of identified pathogens per subject as the outcome and calendar month as the sole predictor, accounting for excess zeros in pathogen counts. Model fit was evaluated via likelihood ratio test against a null zero-inflated model. Finally, pairwise differences in pathogen burden across villages were assessed using Wilcoxon rank-sum tests with Holm correction for multiple comparisons. All analyses were performed in R (version 4.3) using the following packages: tidyverse, gtsummary, pscl, lmtest, rstatix, and ggplot2.

## 3 Results

### 3.1 Distribution of fever cases and burden of pathogens

**Figure 1:**
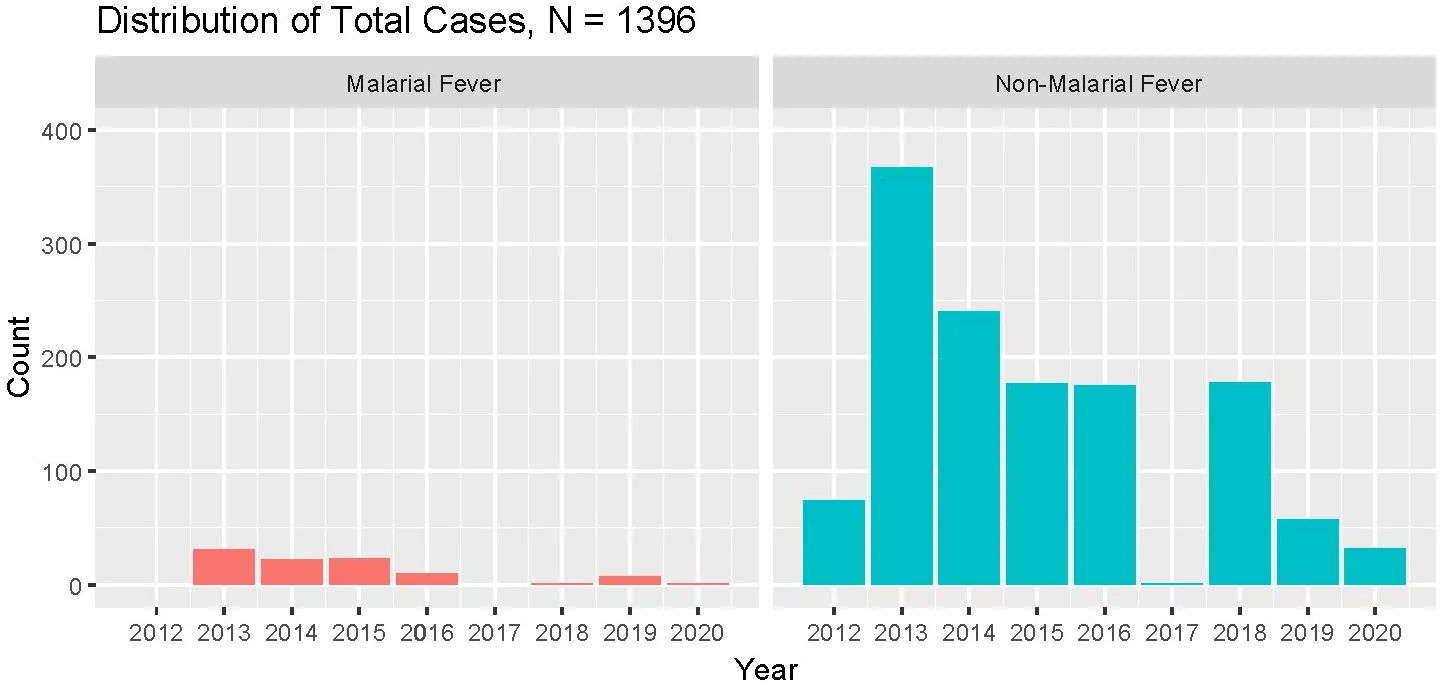
Temporal distribution of febrile illness cases in the full archived sample repository, 2012-2020. Annual counts of febrile illness samples in the full repository (N = 1,396) are shown by malaria status. This figure provides the temporal context for the parent sample archive from which the mNGS-eligible subset was selected.

**Figure 2:**
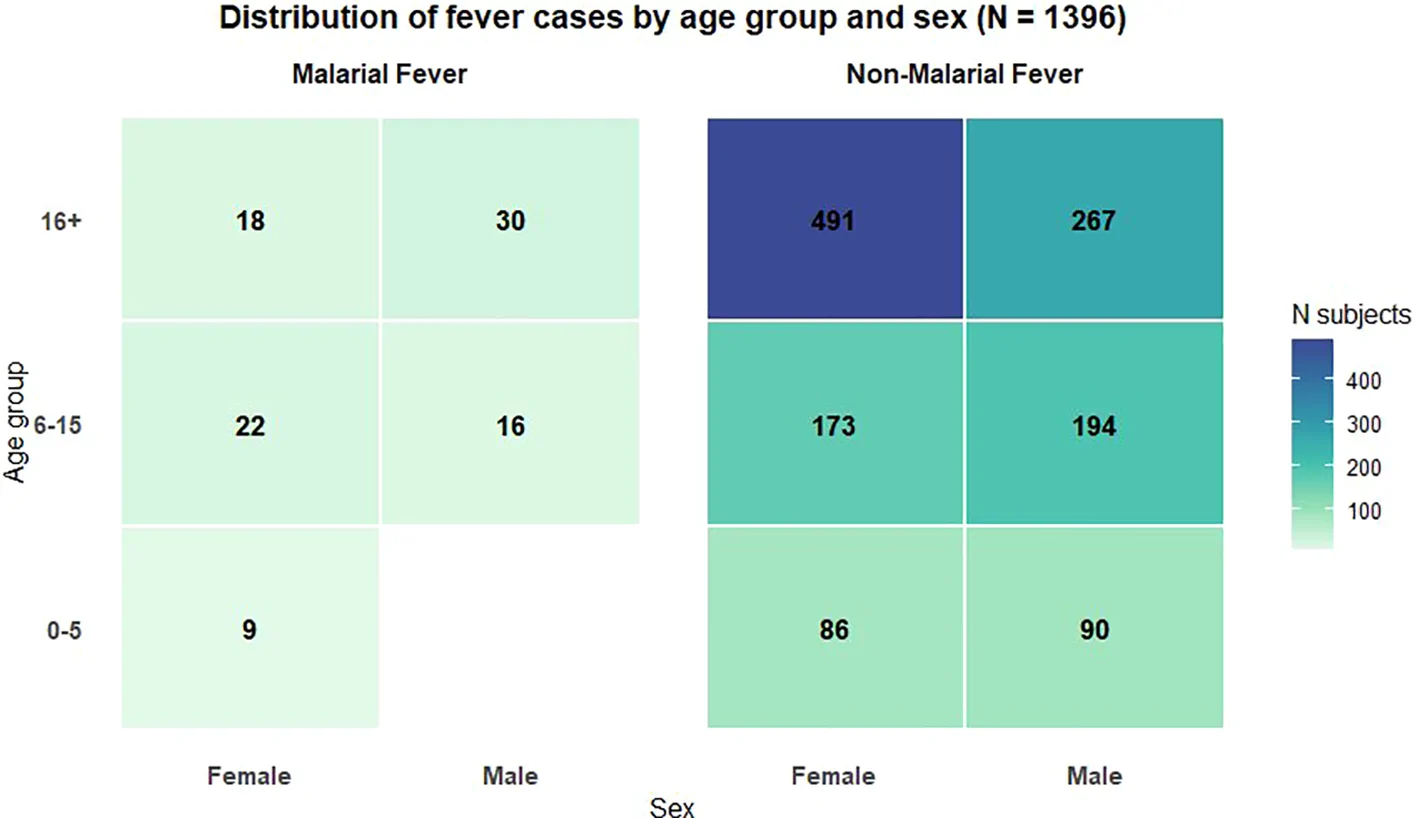
Distribution of febrile illness cases in the full archived sample repository by age group, sex, and malaria status. Counts of malaria-positive and malaria-negative febrile illness cases are shown across age groups (0-5, 6-15, and 16+ years) and sex among all archived samples (N = 1,396). Age groups were selected to reflect young children, school-aged children/adolescents, and adults.

Among the full repository of 1,396 febrile illness samples, 6.80% (95/1396) were malaria positive (malarial fever), while 93.20% (1301/1396) were malaria negative (non-malaria fever). These samples were collected during the period 2012-2020. **Figures 1** and **2** shows the temporal distribution of fever cases by year, and aggregated by age groups (0-5, 6-15, 16+ years old) and sex (female, male). Within the non-malarial fever group, we can observe that burden is higher in the 6–15 and 16+ years old group. Females with 16+ years old represent the group with higher burden of non-malarial fever.

**Figure 3:**
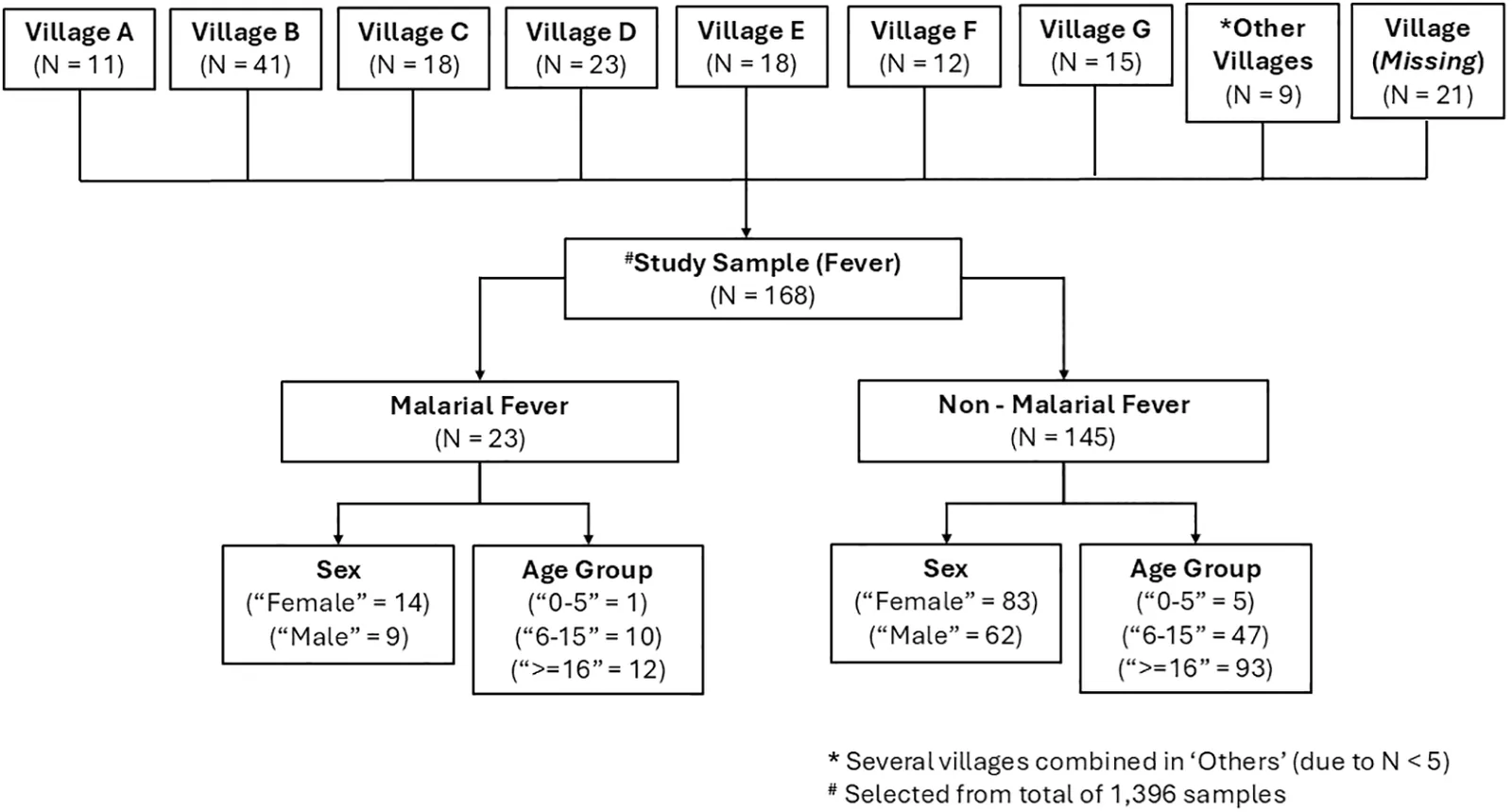
Selection of archived plasma samples eligible for nanopore metagenomic sequencing. Flow diagram shows the selection of the final mNGS analytic cohort from the full archived febrile illness sample repository. Samples were eligible for mNGS if at least 200 μL of archived plasma was available, resulting in 168 sequenced specimens, including 23 malaria-positive and 145 malaria-negative febrile illness samples.

**Figure 4:**
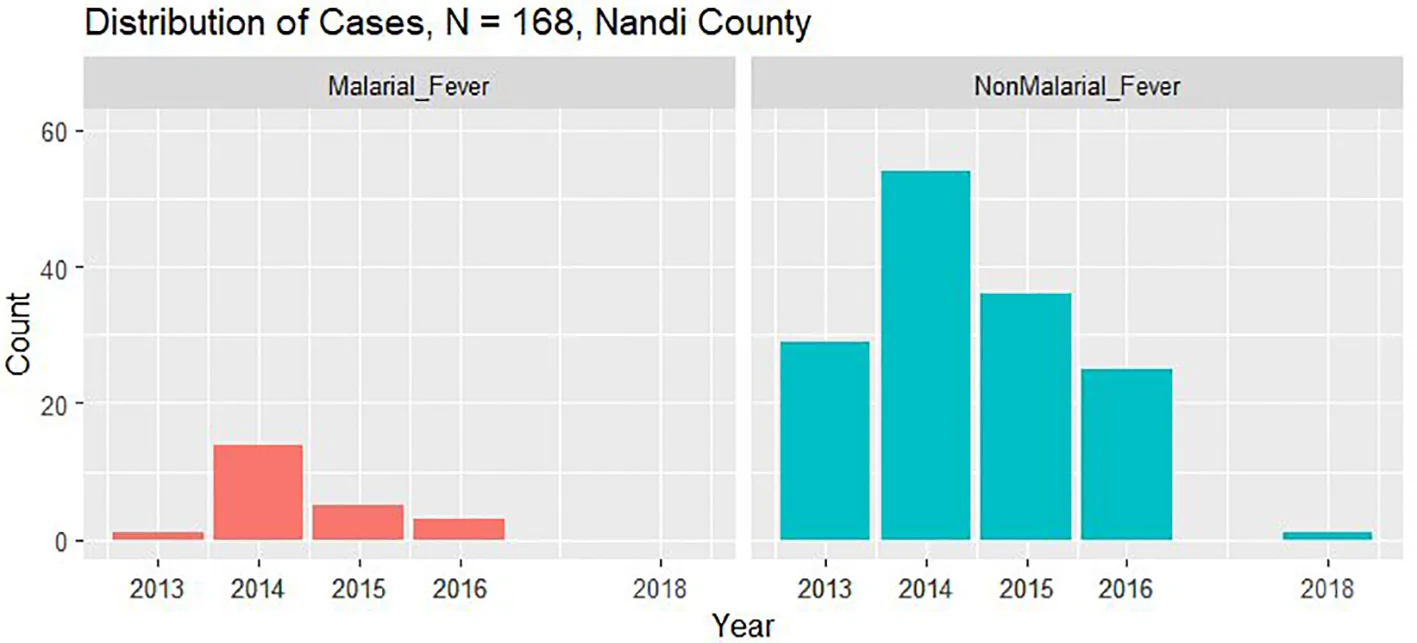
Temporal distribution of febrile illness cases in the mNGS analytic cohort. Annual distribution of the 168 sequenced samples is shown by malaria status. This figure describes the temporal composition of the mNGS subset and should be interpreted in light of plasma-volume-based sample selection (at least 200 μL of archived plasma).

Only those samples meeting plasma volume criteria for metagenomic sequencing were included in the present analysis; however, **Supplementary Material** (**Supplementary Table 3**) presents a simple descriptive table of sequenced vs non-sequenced samples. **Figure 3** illustrates this study sample (N = 168). The final mNGS cohort comprised 23 individuals with malarial fever and 145 individuals with non-malarial fever. Demographic characteristics were broadly comparable between groups (village, sex, age groups). Spatial distribution of fever cases according to malaria infection is provided in **Supplementary Material** (**Supplementary Figure 1**). **Figure 4** shows the temporal distribution of fever cases by year of this selected sample (N = 168).

**Table 1** summarizes the study cohort in detail based on demographic and clinical factors. The cohort was predominantly female (58%) and adult, with 63% aged 16 years or older. Sex and age distribution did not differ significantly between malaria-positive and malaria-negative subjects (p = 0.7 and p = 0.3, respectively). The village of residence was significantly associated with malaria status (p < 0.001); malaria-positive cases were concentrated in Villages A (36%) and C (27%), whereas Villages E, F, and G had no malaria-positive subjects. The majority of subjects resided near a river (94%), and proximity to environmental features did not differ by malaria status (p = 0.6). Malaria-positive subjects lived significantly farther from the nearest health clinic (mean 2.86 km vs. 1.96 km; p < 0.001) and at modestly lower elevations (mean 1,935 m vs. 1,943 m; p = 0.006). Regarding pathogen burden, malaria-positive subjects had a higher mean number of identified pathogens compared to malaria-negative subjects (3.78 vs. 2.14; p = 0.002). This difference was driven primarily by higher counts of opportunistic pathogens (mean 1.91 vs. 0.88; p < 0.001) and common microbiome or environmental contaminants (mean 1.35 vs. 0.52; p = 0.003), while the number of organisms previously reported with potential to cause of febrile illness did not differ significantly between groups (mean 0.52 vs. 0.74; p > 0.9). Confounding and non-random sample selection may affect these descriptive, unadjusted comparisons.

**Table 1:** Descriptive summary of the study sample (N = 168), stratified by malaria status (negative vs positive).

Characteristic	Overall N = 168^1^	Negative N = 145^1^	Positive N = 23^1^	p-value^2^
Sex				0.7
Female	97 (58%)	83 (57%)	14 (61%)	
Male	71 (42%)	62 (43%)	9 (39%)	
Age group				0.3
0-5	6 (3.6%)	5 (3.4%)	1 (4.3%)	
6-15	57 (34%)	47 (32%)	10 (43%)	
16+	105 (63%)	93 (64%)	12 (52%)	
Village				<0.001
A	11 (7.5%)	3 (2.4%)	8 (36%)	
B	41 (28%)	37 (30%)	4 (18%)	
C	18 (12%)	12 (9.6%)	6 (27%)	
D	23 (16%)	21 (17%)	2 (9.1%)	
E	18 (12%)	18 (14%)	0 (0%)	
F	12 (8.2%)	12 (9.6%)	0 (0%)	
G	15 (10%)	15 (12%)	0 (0%)	
Others	9 (6.1%)	7 (5.6%)	2 (9.1%)	
(Missing)	21	20	1	
Proximity to				0.6
Forest	9 (6.1%)	7 (5.6%)	2 (9.1%)	
River	138 (94%)	118 (94%)	20 (91%)	
(Missing)	21	20	1	
Distance to Clinic (km)	2.10; 2.31 (0.95)	1.96; 2.14 (0.95)	2.86; 2.75 (0.46)	<0.001
(Missing)	21	20	1	
Elevation (m)	1,941.88; 1,939.74 (25.49)	1,942.92; 1,941.00 (23.83)	1,935.51; 1,927.01 (33.90)	0.006
(Missing)	4	4	0	
Number of identified pathogens	2.36; 1.00 (4.05)	2.14; 1.00 (3.99)	3.78; 3.00 (4.21)	0.002
Common microbiome or other environmental contaminant	0.64; 0.00 (1.83)	0.52; 0.00 (1.74)	1.35; 0.00 (2.21)	0.003
Established pathogens with potential to cause fever	0.71; 0.00 (2.15)	0.74; 0.00 (2.27)	0.52; 0.00 (1.20)	>0.9
Opportunistic pathogen	1.02; 1.00 (1.30)	0.88; 0.00 (1.24)	1.91; 2.00 (1.35)	<0.001

^1^n (%); Mean; Median (SD).

^2^Pearson’s Chi-squared test; Kruskal-Wallis rank sum test; Fisher’s exact test; Wilcoxon rank sum test.Values in bold indicates p-values that are less than 0.05, considering the variable as statisticial significant at the 5% significance level.

### 3.2 Pathogen diversity detected by mNGS

Across study samples (N = 168), 149 unique microorganisms were detected, including bacteria and viruses. The majority represented known human microbiome constituents (oral, skin, and gut flora), environmental organisms, or ubiquitous viruses (e.g., *Torque teno viruses*). The most prevalent organisms included *Cutibacterium acnes* (n = 7, 4.2%), *Stenotrophomonas maltophilia* (n = 6, 3.6%), *Streptomyces* spp. (n = 5, 3.0%), and *Enterobacter* spp. (n = 4, 2.4%). These organisms span commensal, environmental, and opportunistic categories. Several organisms with established fever-causing potential were detected across multiple individuals, including *Escherichia coli* (n = 3, 1.8%), *Salmonella enterica* (n = 2, 1.2%), *Klebsiella pneumoniae* (n = 2, 1.2%), *Pseudomonas aeruginosa* (n = 3, 1.8%), *Staphylococcus aureus* (n = 2, 1.2%), and *Streptococcus pneumoniae* (n = 2, 1.2%). Opportunistic pathogens such as *Acinetobacter baumannii*, and *Enterococcus faecium* were also identified (both are n = 1, 0.6%). Notably, anaerobic and fastidious organisms with documented invasive potential were detected, including *Veillonella parvula* (n = 3, 1.8%), *Abiotrophia defectiva* (n = 1, 0.6%), and *Granulicatella adiacens* (n = 1, 0.6%). Multiple anelloviruses (*Torque teno virus* and related genotypes) were detected across the cohort (range: n = 1–4, 0.6–2.4%). These viruses are common in human blood and were not interpreted as direct causes of fever but may reflect immune status or systemic inflammation. The full list of pathogens identified by mNGS are provided in **Supplementary Material** (**Supplementary Table 1**), based on the categorization provided in the Methods section.

These organism detections represent taxonomic assignments of microbial nucleic acid in plasma; they do not independently differentiate active infection from transient DNA/RNA, colonization, contamination, or nonviable organisms. Organisms commonly associated with skin, oral flora, reagents, or the environment were therefore classified as background or potential contaminants, unless supported by clinical plausibility and known invasive potential.

**Figure 5:**
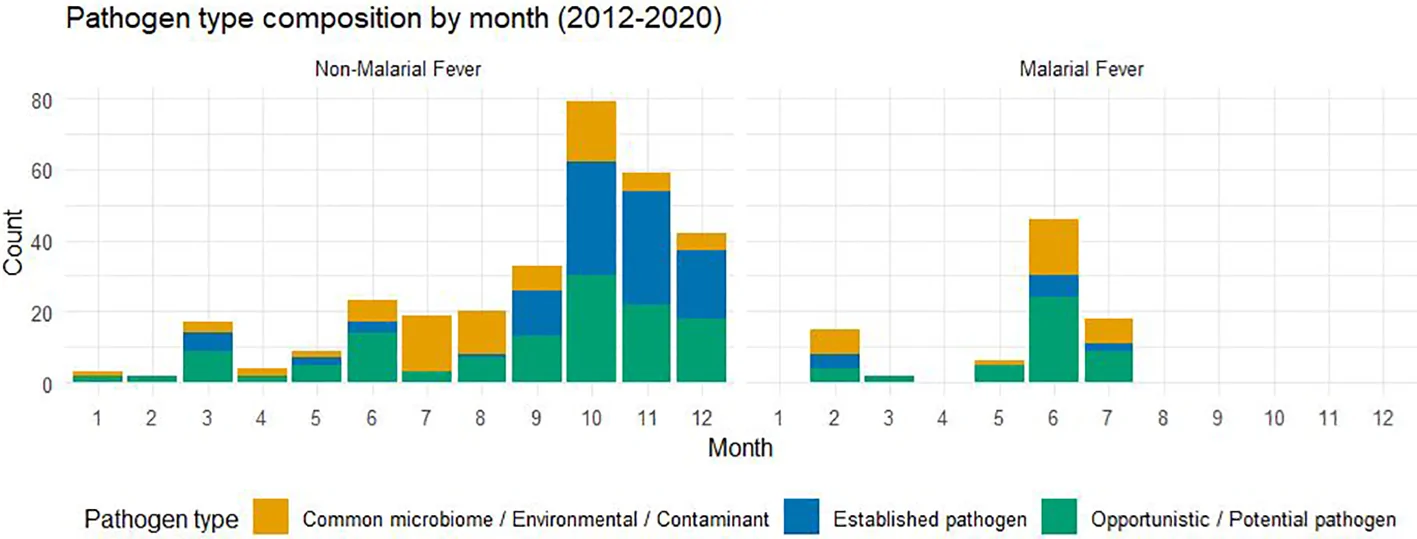
Monthly composition of detected microbial taxa by fever group and interpretation category. Detected organisms are summarized by calendar month and fever group, stratified by interpretation category, including common microbiome/environmental taxa, opportunistic organisms, and organisms with established fever-causing potential. Counts represent taxonomic detections from mNGS.

**Figure 6:**
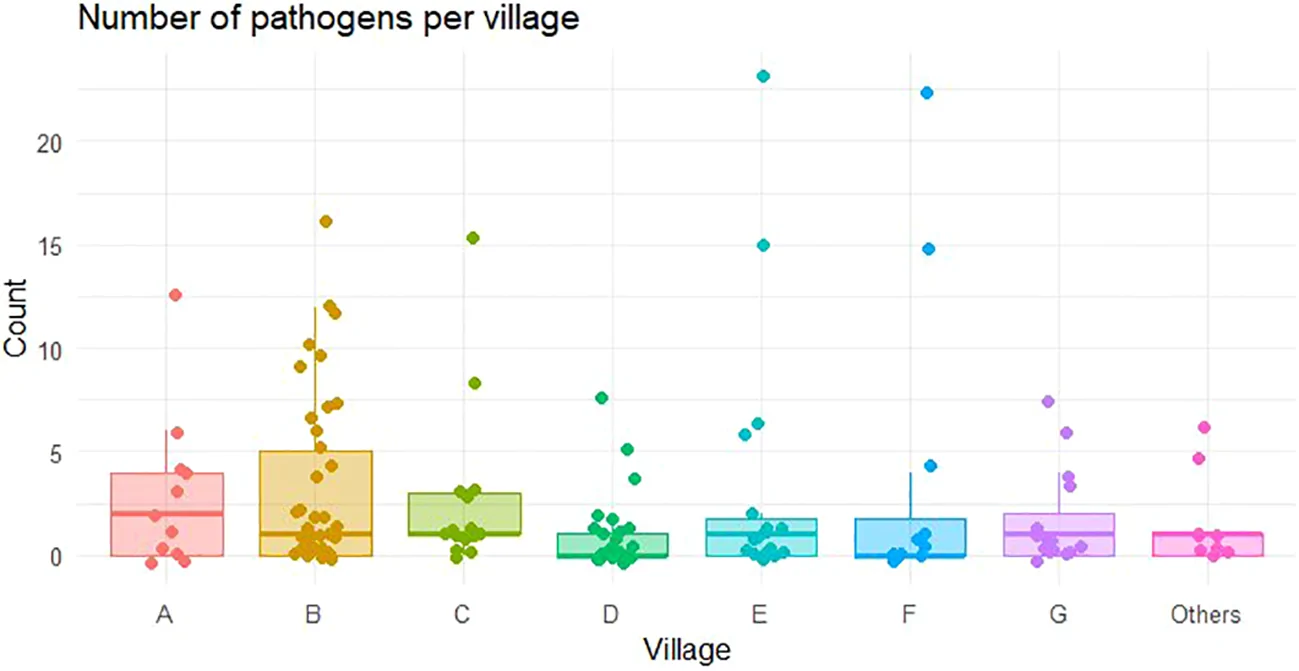
Distribution of the number of detected microbial taxa per individual by village. Boxplots show the number of detected microbial taxa per sequenced individual across villages. Points represent individual participants.

**Figure 5** shows the pathogen type composition by overlapping month during the period 2012–2020 and stratified by fever group. Among non-malarial fever cases, pathogen counts peaked in October and November, with opportunistic/potential pathogens and established fever-causing pathogens each contributing substantially during these months. Common microbiome or environmental contaminants were most prominent in July and August. Among malarial fever cases, detections were concentrated in February and May–July, with opportunistic and environmental organisms comprising the majority of identified pathogens. Months outside these windows had minimal or no detections in both groups. When looking at the number of pathogens by village (**Figure 6**), the number of identified pathogens varied, with Villages A, B, and C showing the highest median counts and greatest spread. Villages D, E, F, G, and Others had consistently lower medians, though all villages exhibited high-count outliers, reflecting the skewed distribution of pathogen burden across subjects. After performing multiple comparisons in the number of pathogens identified by village (before and after adjustment with Benjamini-Hochberg correction), no statistical differences were found.

### 3.3 Regression analyses and likelihood ratio test

Univariable and multivariable logistic regression models were used to examine predictors of non-malarial fever (**Table 2**). In both analyses, calendar month (modeled linearly) was a significant positive predictor, with the odds of non-malarial fever increasing with each successive month (OR = 1.26, 95% CI: 1.09 - 1.48, p = 0.002; adjusted OR [aOR] = 1.42, 95% CI: 1.15 - 1.81, p < 0.001). We address limitations of this specification in the Discussion section. Distance to clinic was significantly and inversely associated with non-malarial fever in both models, indicating that subjects residing farther from a health facility had lower odds of a non-malarial diagnosis (OR = 0.217, 95% CI: 0.088 - 0.453, p < 0.001; aOR = 0.241, 95% CI: 0.088 – 0.548, p < 0.001). Age showed a trend toward association in both models but did not reach statistical significance (OR 1.02, p = 0.055; aOR 1.03, p = 0.057). Sex and household elevation were not significantly associated with fever etiology in either model (p = 0.743 and p = 0.407 in univariable model; p = 0.120 and p = 0.161 in the multivariable model, respectively).

**Table 2:** Logistic regression analyses for fever group indication (0 = malarial fever vs 1 = non-malarial fever).

Characteristic	Univariable				Multivariable		
	N	OR	95% CI	p-value	aOR	95% CI	p-value
**Month**	168	1.262	1.088, 1.478	**0.002**	1.421	1.154, 1.806	**<0.001**
Sex	168			0.743			0.407
Female		—	—		—	—	
Male		1.162	0.478, 2.952		1.590	0.535, 5.021	
Age	168	1.024	1.000, 1.053	0.055	1.033	0.999, 1.074	0.057
Distance to clinic (km)	147	0.217	0.088, 0.453	<0.001	0.241	0.088, 0.548	<0.001
Household elevation (m)	164	1.015	0.995, 1.039	0.161	1.022	0.995, 1.056	0.120

CI, Confidence Interval; OR, Odds Ratio; aOR, Adjusted Odds Ratio.Values in bold indicates p-values that are less than 0.05, considering the variable as statisticial significant at the 5% significance level.

Likelihood ratio tests (LRT) confirmed that calendar month significantly improved model fit beyond the null model. When non-malarial fever status was the outcome, adding month to the logistic regression model yielded a significant improvement in fit (LRT = 9.50, df = 1, p = 0.002), consistent with the regression findings above. Similarly, when the number of identified pathogens per subject was modeled using a zero-inflated Poisson regression, month again contributed significantly to model fit (LRT = 20.67, df = 2, p < 0.001).

## 4 Discussion

This study demonstrates the feasibility of nanopore mNGS for exploratory characterization of microbial nucleic-acid signatures in archived plasma specimens from febrile patients in Western Kenya. The findings reveal a heterogeneous mixture of common microbiome/environmental taxa, opportunistic organisms, ubiquitous viruses, and organisms with established fever-causing potential.

Specifically, the study assess the relationships between malaria co-infections, microbiome alterations, and ubiquitous viruses in febrile patients, demonstrating that understanding fever etiology and temporal variations is vital for improving diagnosis, treatment, and surveillance in the context of emerging and re-emerging infections (Fares, 2013). These findings align with broader evidence from sub-Saharan Africa showing that NMFI is driven by diverse bacterial and viral pathogens often undetectable through routine diagnostics (Prasad et al., 2015a). The detection of *Escherichia coli*, *Klebsiella pneumoniae*, *Salmonella enterica*, *Pseudomonas aeruginosa*, and *Staphylococcus aureus* in multiple individuals reflects patterns seen in Kenyan hospital-based surveillance, where invasive bacterial infections remain common (Verani et al., 2024). While malaria remains a primary diagnostic target in endemic regions, our study underscores the critical burden of co-infections. Failure by healthcare facilities to investigate beyond a positive malaria test leads to incomplete treatment regimens, ultimately driving poor clinical outcomes (Wilairatana et al., 2022; Cunnington et al., 2024). While detected microbial profiles varied by calendar month, these exploratory patterns suggest temporal heterogeneity in microbial detections, but they do not by themselves establish co-infection or fever causality.

An important observation from this study is the temporal heterogeneity in pathogen detection across the study period. The abundance of pathogens did not consistently overlap with periods in which malaria-positive febrile illness was more frequently observed. Instead, opportunistic pathogens and established organisms demonstrated distinct monthly peaks, particularly during October and November among non-malarial fever cases. These findings suggest that additional environmental, ecological, or vector-related drivers may contribute to fever patterns independently of malaria transmission. Seasonal fluctuations in infectious diseases are influenced by rainfall, humidity, vector ecology, human behavior, and population movement, all of which can alter pathogen circulation patterns (Fares, 2013). Similar observations have been reported in rural African settings where many febrile illnesses initially presumed to be malaria were ultimately associated with alternative infectious etiologies (Baltzell et al., 2019). The temporal mismatch observed in our data further reinforces the concern that relying solely on malaria diagnostics may obscure concurrent or unrelated infectious processes.

The detection of opportunistic and environmentally associated organisms, including *Stenotrophomonas maltophilia*, *Acinetobacter baumannii*, and *Enterococcus faecium*, may reflect environmental exposure, transient circulating microbial nucleic acid, healthcare-associated acquisition, host susceptibility, or contamination. These findings identify organisms that warrant targeted follow-up but cannot confirm invasive disease without orthogonal testing. Nanopore mNGS also detected fastidious anaerobic and nutritionally variant organisms such as *Veillonella parvula*, *Granulicatella adiacens*, and *Abiotrophia defectiva*, all of which have recognized invasive potential but rarely appear in conventional culture workflows. These findings reinforce the capability of mNGS to reveal clinically meaningful pathogens that are systematically missed by standard diagnostic modalities. In this study, we observed distinct microbiome-associated profiles among febrile individuals, supporting the concept that fever etiology may involve interactions between host immunity, microbial composition, and pathogen exposure. Although organisms categorized as commensal or non-pathogenic rarely cause fever independently, immune activation triggered by pyrogens, inflammation, or concurrent infections may contribute substantially to febrile responses (El-Radhi, 2018). These observations underscore the importance of detailed fever histories and broader diagnostic evaluation in resource-limited settings where laboratory infrastructure remains constrained. In the absence of advanced laboratory infrastructure, integrating clinical fever trajectories with microbiome insights could provide a valuable, accessible tool for risk stratification and therapeutic decision-making (Bongers et al., 2023; Nagai et al., 2023).

The clinical significance of *anellovirus* detection remains uncertain, although previous studies have suggested potential associations with immune status (Sabbaghian et al., 2024). The present study was not designed to evaluate that relationship. The detection of *anelloviruses* should be interpreted cautiously. These viruses are highly prevalent in human populations and are not generally considered direct causes of acute febrile illness. Future studies incorporating immune phenotyping, quantitative viral-load measures, and longitudinal sampling would be needed to determine whether *anellovirus* detection has clinical or immunologic significance in this setting. Ubiquitous viruses, which are established among the most frequent etiology of acute febrile episodes in the general population, can serve as evolutionary reservoirs for continuous viral replication and the generation of novel, immune-evasive variants (Raglow and Lauring, 2025; Craig et al., 2026). Our results mirror studies from Uganda, Brazil, and West Africa, where nanopore-based sequencing was successfully employed to uncover pathogens during outbreaks, including Ebola, Zika, and Lassa fever, and to resolve undiagnosed febrile illness cases (Faria et al., 2016; Quick et al., 2016; Kafetzopoulou et al., 2019; Kairu-Wanyoike et al., 2019; Di Paola et al., 2020; Levine et al., 2024; Mandai et al., 2024; Aceng et al., 2025; Ashraf et al., 2025). The concordance between our findings and these reports highlights the growing relevance of mNGS as a frontline epidemiological tool in low-resource settings. In particular, nanopore’s portability and rapid sequencing make it a strong candidate for integration into national surveillance platforms such as Kenya’s Population-Based Integrated Disease Surveillance (PBIDS) network, where febrile illnesses are one of their priority surveillance areas (KEMRI, 2006). The distinct temporal variation in fever etiology observed at our study site emphasizes the critical importance of implementing structured, year-round characterization frameworks in low-resource settings, where the absence of such longitudinal data routinely leads to the systematic overdiagnosis of malaria and the inappropriate use of empiric antimicrobials (Reyburn et al., 2004; Aceng et al., 2025).

The observed association between distance to clinic and NMFI status may also reflect broader structural and environmental determinants of febrile illness. Individuals living farther from healthcare facilities may experience delayed care-seeking, reduced diagnostic access, inconsistent treatment adherence, or greater exposure to environmental risk factors. Similar geographic barriers to healthcare utilization have been documented in other low-resource settings and may contribute to differences in disease burden and diagnostic outcomes (Seidu, 2021; Cox et al., 2023). Additionally, the possibility that mosquito vectors traditionally associated with malaria transmission may simultaneously contribute to the circulation of other vector-borne pathogens warrants consideration. Several *Anopheles* species have been implicated in the transmission of non-malarial pathogens capable of causing febrile syndromes, suggesting that shifts in vector ecology may alter the broader epidemiology of febrile illness beyond malaria alone (Onen et al., 2023).

Several limitations should be acknowledged. First, revisiting the operational definition of fever should be prioritized in future studies. In the current study, febrile illness was defined primarily by body temperature without incorporating symptom duration, recurrence patterns, or accompanying clinical manifestations. Refining fever phenotypes may improve etiologic classification and interpretation. Second, as with all DNA-based mNGS procedures, detection of microbial sequences does not distinguish between active infection, transient circulating DNA, colonization, or contamination. The detection of common skin-associated organisms and environmental contaminants likely reflects, in part, unavoidable contamination introduced during venipuncture or specimen handling. Human skin remains a major source of blood culture contamination because antiseptic procedures cannot eliminate all microorganisms residing within deeper skin layers (Caldeira et al., 2011; Bunn and Cornish, 2025). Consequently, some organisms categorized as common microbiome or environmental contaminants should be interpreted cautiously. The lack of paired pathogen-specific diagnostics further limits causal inference between detected organisms and clinical fever. Additionally, the limited sample size, particularly among malaria-positive subjects, restricts robust comparative analyses. Another major limitation is the absence of batch-matched negative extraction controls and no-template controls for this retrospective analysis of archived samples. As a result, formal background subtraction could not be performed, and common skin, oral, reagent-associated, and environmental taxa may represent contamination rather than biologically meaningful infection. The analysis also relied on EPI2ME report outputs rather than raw read-level reassignment files, limiting the ability to calculate genome-wide coverage or depth, evaluate batch effects in detail, or apply customized contaminant filtering after the fact. Future work should include negative extraction controls and no-template controls in every batch, explicit host-read exclusion, raw read-assignment output, a customized database of clinically relevant pathogen that excludes common contaminants, and confirmatory targeted assays for organisms of clinical interest. Furthermore, we recognize that the linear month effect specification considered in this study does not fully capture cyclic seasonality; therefore, results are interpreted as exploratory evidence of temporal patterns rather than as a definitive seasonal model, and future work will assess this appropriately. Although the laboratory protocol included bacterial, viral, and fungal nucleic-acid preparation, this analysis did not utilize a dedicated parasitic pathogen workflow beyond the malaria status ascertained in the parent study. Therefore, protozoan causes of fever, such as *Leishmania* spp. or others, may have been missed. Lastly, we recognize that fever etiologies and the clinical interpretation of microbial detections may vary within the chosen age groups, particularly among infants and very young children. However, the mNGS analytic cohort was limited in size, and more granular pediatric age bands, such as 0–1 years, would have resulted in sparse strata. Therefore, age-stratified results should be interpreted descriptively, and future larger studies should evaluate finer pediatric age categories.

Nevertheless, these findings provide important groundwork for future investigations exploring the contribution of opportunistic pathogens, anaerobes, microbiome alterations, and overlooked viral agents to NMFI in sub-Saharan Africa.

## 5 Conclusion

In conclusion, nanopore-based mNGS provided an exploratory view of microbial nucleic-acid signatures among febrile hospitalized patients in a western Kenya highland setting with declining malaria transmission. While highlighting the importance of non-malarial and concurrent infections, organism detection requires cautious interpretation due to limited contamination controls and confirmatory diagnostics. Transforming these exploratory insights into clinical utility requires future prospective studies that integrate systematic sampling frameworks, field-deployable contamination controls, and causal confirmation methods to safely guide regional antimicrobial stewardship and digital surveillance.

## Data Availability

The data that supports the findings of this study are available within the manuscript. The raw data is not publicly available due to privacy and ethical restrictions, as they contain information that could compromise the privacy of human research participants. Summarized, de-identified data can be made available by the corresponding author upon reasonable request.
