# Supplementary material for: Using nanopore metagenomics to characterize pathogens in febrile patients from a highland of Western Kenya
Source: Front Cell Infect Microbiol. 2026 Aug 12;16:1884846. doi: 10.3389/fcimb.2026.1884846 (PMC13506779; doi:10.3389/fcimb.2026.1884846)
Supplement: Supplementary file 1 [file Presentation1.pdf]

Supplementary Material

Using nanopore metagenomics to characterize pathogens in febrile patients from a highland of Western Kenya

Felix Pabon-Rodriguez*[1#] and George Ayodo[2#]

[1]Department of Biostatistics and Health Data Science, Indiana University School of Medicine, Indianapolis, Indiana, United States

[2]Centre for Community Health and Wellbeing, Jaramogi Oginga University of Science and Technology, Bondo, Kenya

[#]Authors contributed equally

Correspondence

*Felix M. Pabon-Rodriguez

Email: fpabonrodriguez@gmail.com

https://orcid.org/0000-0003-3528-2354

1 Supplemental Figures and Tables

Figure S1. Spatial distribution of febrile illness cases by malaria status in the mNGS analytic cohort. Household locations of sequenced participants are mapped by malaria status, with village boundaries shown for geographic context. This figure illustrates the spatial distribution of the mNGS subset and should not be interpreted as population-level village incidence without village denominator data.

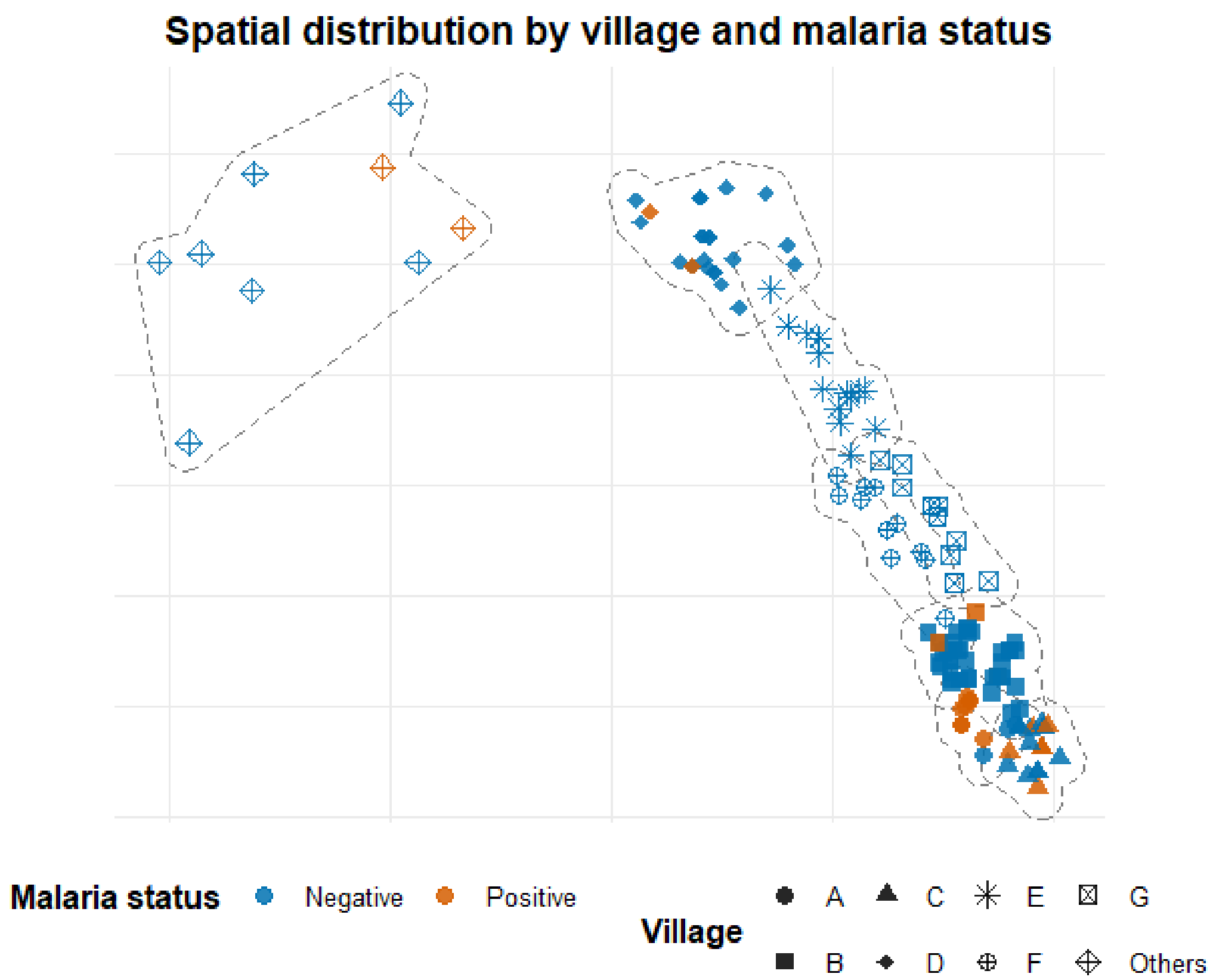

Table S1. Full list of microorganisms detected by mNGS, prevalence, and category.

(a) Established pathogen

Organism	*Individuals (n)*
Bacillus cereus	4
Clostridium botulinum	4
Klebsiella pneumoniae	3
Pseudomonas aeruginosa	3
Streptococcus gordonii	3
Streptococcus oralis	2
Escherichia coli	2
Bacillus velezensis	2
Streptococcus mutans	2
Fictibacillus phosphorivorans	2
Pseudomonas sp. Colony2	2
Staphylococcus ureilyticus	1
Streptococcus sp. LPB0220	1
Streptococcus sp. DTU_2020_1001019_1_SI_AUS_MUR_006	1
Streptococcus sp. DTU_2020_1000888_1_SI_GRL_NUU_041A	1
Streptococcus sp. A12	1
Streptococcus salivarius	1

Streptococcus pneumoniae	1
Streptococcus parasuis	1
Streptococcus mitis	1
Streptococcus cristatus	1
Clostridium sp. C45	1
Staphylococcus cohnii	1
Staphylococcus aureus	1
Bacillus thuringiensis	1
Clostridium tetani	1
Streptococcus vestibularis	1
Clostridium intestinale	1
Acinetobacter sp. TTH0-4	1
Acinetobacter baumannii	1
Mycobacteroides chelonae	1
Fictibacillus arsenicus	1
Streptococcus sp. Marseille-Q3533	1
Pseudomonas sp. DTU_2021_1001937_2_SI_NGA_ILE_001	1
Streptococcus sanguinis	1
Pseudomonas putida	1
Pseudomonas sp. S35	1

Proteus mirabilis	1
Pseudomonas veronii	1
Pseudomonas beijingensis	1
Pseudomonas syringae	1
Pseudomonas chlororaphis	1
Pseudomonas sp. p1(2021b)	1
Pseudomonas coleopterorum	1
Pseudomonas cremoricolorata	1
Pseudomonas sp. MM211	1
Salmonella enterica	1
Pseudomonas protegens	1
Pseudomonas fluorescens	1
Pseudomonas fulva	1
Pseudomonas fuscovaginae	1
Pseudomonas sp. Leaf58	1
Pseudomonas germanica	1
Pseudomonas oryzihabitans	1
Pseudomonas parafulva	1
Pseudomonas entomophila	1
Enterococcus faecium	1

(b) Opportunistic pathogen

Organism	*Individuals (n)*
Cutibacterium acnes	7
Stenotrophomonas maltophilia	6
Enterobacter sp. T2	4
Veillonella parvula	3
Staphylococcus epidermidis	2
Pseudomonas sp. CIP-10	2
Acinetobacter johnsonii	1
Cutibacterium granulosum	1
Finegoldia magna	1
Granulicatella adiacens	1
Haemophilus parainfluenzae	1
Leptotrichia wadei	1
Neisseria macacae	1
Neisseria sicca	1
Neisseria subflava	1
Acinetobacter lwoffii	1
Capnocytophaga sputigena	1
Staphylococcus capitis	1

Staphylococcus hominis	1
Schaalia odontolytica	1
Veillonella atypica	1
Abiotrophia defectiva	1
Rothia mucilaginosa	1
Cutibacterium avidum	1

(c) Common microbiome / Environmental / Contaminant

Organism	*Individuals (n)*
Streptomyces sp. T12	5
Halomonas sp. JS92-SW72	3
Corynebacterium kefirresidentii	3
Corynebacterium matruchotii	2
Sphingobacterium spiritivorum	2
Sphingobacterium sp. UDSM-2020	2
Prevotella melaninogenica	2
Anelloviridae sp.	2
Hydrogenophaga sp. NH-16	2
Comamonas sp. B21-038	1
Olivibacter sp. LS-1	1

Olivibacter sp. SDN3	1
Leuconostoc gelidum	1
Pahexavirus PHL009M11	1
Corynebacterium doosanense	1
Alphatorquevirus homin13	1
Actinomyces sp. oral taxon 414	1
Alphatorquevirus homin10	1
Nodularia sphaerocarpa	1
Alphatorquevirus homin18	1
Alphatorquevirus homin20	1
Olivibacter sp. EAT-5	1
Megamonas hypermegale	1
Moraxella osloensis	1
Microbacterium oxydans	1
Methylorubrum populi	1
Alphatorquevirus homin3	1
Megamonas funiformis	1
Massilia oculi	1
Corynebacterium propinquum	1

Lawsonella clevelandensis	1
Lactococcus cremoris	1
Kluyvera ascorbata	1
Gluconacetobacter diazotrophicus	1
Gemella morbillorum	1
Gammatorquevirus homidi15	1
Corynebacterium tuberculostearicum	1
Alphatorquevirus homin29	1
Aurantimicrobium sp. MWH-Uga1	1
Alphatorquevirus homin5	1
Pantoea anthophila	1
Rhizobium sp. X9	1
Wolbachia endosymbiont of Drosophila pseudotakahashii	1
Torque teno virus 8	1
Torque teno virus 23	1
Torque teno virus	1
Thermoanaerobacterium thermosaccharolyticum	1
TTV-like mini virus	1
Spirosoma pollinicola	1

Pantoea sp. MT58	1
Sphingobacterium multivorum	1
Sediminibacterium sp. KACHI17	1
Prevotella histicola	1
Comamonas aquatica	1
Anaerococcus sp. WGS1579	1
Candidatus Southlakia epibionticum	1
Brachybacterium sp. GU-2	1
Brachybacterium sp. GPGPB12	1
Blautia pseudococcoides	1
Bifidobacterium dentium	1
Betatorquevirus homini8	1
Betatorquevirus homini7	1
Bacteroides uniformis	1
Bacteroides caecimuris	1
Aureimonas altamirensis	1
Corynebacterium accolens	1
Anaerocolumna chitinilytica	1
Xanthomonas albilineans	1

2 Pathogen Metagenomic Sequencing Protocol

2.1 Metagenomic Sequencing

Metagenomic sequencing was performed to identify bacterial, fungal, and viral pathogens in plasma samples using the Rapid PCR Barcoding Kit V14 (SQK-RPB114.24) and the RMS_9215_v114_revF_18Nov2025 protocol from Oxford Nanopore Technologies.

For each patient, 200 µL of plasma was centrifuged at 10,000 × g for 5 minutes. The supernatant was transferred to a fresh tube for viral nucleic acid extraction, and the pellet was reserved for bacterial and fungal DNA isolation.

2.2 Viral Nucleic Acid Extraction and Reverse Transcription

2.2.1 Host Nucleic Acid Depletion

To reduce background host nucleic acids, 10 µL of HL-SAN endonuclease (70910202; ArcticZymes Technologies) was added to the plasma supernatant and incubated at 37°C for 10 minutes at 1,000 rpm.

2.2.2 Nucleic Acid Extraction

Viral nucleic acids were extracted using the MagMAX Viral/Pathogen Ultra Nucleic Acid Isolation Kit (A42356; Applied Biosystems) according to the manufacturer's instructions. Briefly, 10 µL Proteinase K, 520 µL Binding Buffer, and 20 µL magnetic beads were added to each sample and incubated at 65°C for 5 minutes at 1,000 rpm, followed by 5 minutes at room temperature. Nucleic acids were eluted in 15 µL nuclease-free water.

2.2.3 Reverse Transcription

Reverse transcription was performed using RLB RT 9N primer (5′-TTTTTCGTGCGCCGCTTCAACNNNNNNNNN-3′), TSOmG template-switching oligonucleotide (5′-GCTAATCATTGCTTTTTCGTGCGCCGCTTCAACATmGmGmG-3′), and Maxima H Minus Reverse Transcriptase (13233159; Thermo Fisher Scientific). Reactions were incubated at 42°C for 75 minutes, followed by enzyme inactivation at 80°C for 5 minutes.

2.3 Bacterial and Fungal DNA Isolation

The plasma pellet was resuspended in 200 µL of 0.2% saponin in phosphate-buffered saline (PBS). Subsequently, 200 µL of 2× HL-SAN buffer and 10 µL HL-SAN enzyme were added, and samples were incubated at 37°C for 10 minutes.

After addition of 900 µL PBS, samples were centrifuged at 12,000 × g for 3 minutes. The supernatant was discarded, and the pellet was resuspended in 200 µL PBS.

DNA extraction was performed using the MagMAX Viral/Pathogen Ultra Nucleic Acid Isolation Kit. Briefly, 50 µL Enzyme Mix was added and incubated at 65°C for 15 minutes, followed by addition of 10 µL Proteinase K, 530 µL Binding Buffer, and 20 µL magnetic beads. Samples were incubated at 65°C for 5 minutes, and DNA was eluted in 20 µL nuclease-free water.

2.4 Library Preparation

2.4.1 Tagmentation

Bacterial/Fungal DNA fragmentation was performed using Fragmentation Mix (FRM) from the Rapid Barcoding Kit (Oxford Nanopore Technologies). Three microliters of DNA eluate were combined with 1 µL FRM and incubated at 30°C for 2 minutes, 80°C for 2 minutes, and held at 10°C.

2.4.2 Rapid Barcode PCR

For each reaction, 5 µL of reverse transcription product and 5 µL of purified bacterial/fungal DNA were combined with 25 µL LongAmp Hot Start Taq 2× Master Mix (NEB, M0533), 11 µL nuclease-free water, and 10 µL Rapid Barcode Primers (SQK-RPB114.24; Oxford Nanopore Technologies).

PCR conditions were as follows: initial denaturation at 95°C for 45 seconds; 30 cycles of 95°C for 15 seconds, 56°C for 15 seconds, and 65°C for 4 minutes. Subsequently, 1 µL Thermolabile Exonuclease I was added to each reaction and incubated at 37°C for 10 minutes and 80°C for 1 minute.

PCR products were pooled, and the barcoded DNA library was purified using AMPure XP beads (Oxford Nanopore Technologies).

2.5 Sequencing and Bioinformatic Analysis

The pooled barcoded libraries were sequenced on a MinION platform using R10.4.1 flow cells (FLO-MIN114) from Oxford Nanopore Technologies.

Basecalling was performed using MinKNOW (version 25.09.16). Taxonomic classification and metagenomic analysis were conducted using the EPI2ME wf-metagenomic workflow.

3 EPI2ME Report Details / QC Values

Table S2: Report details from EPI2ME.

Metric	Value from EPI2ME reports
Workflow/report version	wf-metagenomics report v2.13.0; report date shown as 2026-01-29
Classifier/database	classifier = kraken2; database_set = Standard-8; taxonomic_rank = S
Tool versions	Kraken 2.1.3; minimap2 2.28-r1209; samtools 1.21; fastcat 0.20.0; taxonkit v0.19.0; pysam 0.23.0; pandas 2.2.3
Key thresholds/settings	min_percent_identity = 90; min_ref_coverage = 0; abundance_threshold = 0; kraken2_confidence = 0; min_len = 0
Host handling	Laboratory host depletion used HL-SAN. Report parameters do not document host-exclusion as enabled (`exclude_host` blank; `minimap2exclude` false), and Homo sapiens appears in taxonomy tables.
Read assignments	include_read_assignments = False
Coverage/depth	No barcode-level genome coverage/depth summary was evident in the HTML reports.

Read metric across 168 barcodes	Median	Mean	Range
Total reads	4,237.5	12,692.5	174 to 192,132
Unclassified/unmapped reads	2,545.5	5,903.2	81 to 54,168
Unclassified/unmapped percentage	78.31%	65.59%	1.20% to 99.43%
Classified/mapped reads	801.0	6,789.3	5 to 182,670
Classified/mapped percentage	21.70%	34.41%	0.57% to 98.80%

Host nucleic acid depletion was performed experimentally using HL-SAN before extraction. The EPI2ME reports did not provide sufficient evidence to confirm complete computational host-read exclusion for all runs; therefore, host-associated reads were not used for microbial interpretation. Future analyses will use explicit host-read depletion/exclusion settings and a customized clinically relevant pathogen database that excludes common skin, reagent, and environmental organisms.

Barcode-level QC metrics were extracted from the EPI2ME reports, including total reads, unclassified/unmapped reads, and classified/mapped reads. Across 168 barcodes, the median total read count was 4,237.5 reads (range, 174-192,132), the median unclassified/unmapped percentage was 78.31% (range, 1.20%-99.43%), and the median classified/mapped read count was 801 reads

(range, 5-182,670). The reports did not contain sufficient genome coverage/depth summaries to support variant or SNP analysis, and no variant detection was attempted.

4 Comparison Table: Sequenced vs Non-Sequenced Samples

Table S3: Descriptive summary of sequenced (Yes) vs non-sequenced samples (No).

Characteristic	Overall<br>N = 1,397[1]	No<br>N = 1,229[1]	Yes<br>N = 168[1]
**Sex**			
Female	799 (57%)	702 (57%)	97 (58%)
Male	597 (43%)	526 (43%)	71 (42%)
(Missing)	1	1	0
**Age Group**			
0-5	185 (13%)	179 (15%)	6 (3.6%)
6-15	405 (29%)	348 (28%)	57 (34%)
16+	806 (58%)	701 (57%)	105 (63%)
(Missing)	1	1	0
**Malaria**			
Negative	1,301 (93%)	1,156 (94%)	145 (86%)
Positive	95 (6.8%)	72 (5.9%)	23 (14%)
(Missing)	1	1	0
**Blood volume (µL)**	85.50; 75.00 (56.10)	69.23; 75.00 (35.32)	204.46; 200.00 (30.63)
(Missing)	1	1	0

[1]n (%); Mean; Median (SD)

5 Operational criteria for pathogens

Category	Operational definition[1–20]
Common microbiome/environmental/possible contaminant	Organisms primarily known as skin, oral, gastrointestinal, soil, water, plant, reagent-associated, or laboratory-environment taxa.
Opportunistic pathogen	Organisms with documented pathogenic potential under specific host, exposure, hospitalization, device, mucosal-barrier, or immunosuppression contexts.
Established pathogen	Organisms with well-established capacity to cause invasive, bloodstream, enteric, respiratory, urinary, toxin-mediated, or systemic febrile illness in humans.
Common virome/background signal	Ubiquitous human virome members or viral/bacteriophage signals without demonstrated direct fever causality in this study.

References:

1. Kaczorowska J, van der Hoek L. Human anelloviruses: diverse, omnipresent and commensal members of the virome. FEMS Microbiol Rev. 2020 May 1;44(3):305–13. doi:10.1093/femsre/fuaa007

2. Spandole S, Cimponeriu D, Berca LM, Mihăescu G. Human anelloviruses: an update of molecular, epidemiological and clinical aspects. Arch Virol. 2015 Apr 15;160(4):893–908. doi:10.1007/s00705-015-2363-9

3. Dumm RE, Wing A, Richterman A, Jacob J, Glaser LJ, Rodino KG. The Brief Case: A Variant on a Classic—Abiotrophia defectiva Endocarditis with Discitis. J Clin Microbiol. 2021 Sep 20;59(10). doi:10.1128/JCM.03093-20

4. Marriott D, Stark D, Harkness J. *Veillonella parvula* Discitis and Secondary Bacteremia: a Rare Infection Complicating Endoscopy and Colonoscopy? J Clin Microbiol. 2007 Feb;45(2):672–4. doi:10.1128/JCM.01633-06

5. Davin-Regli A, Lavigne JP, Pagès JM. *Enterobacter* spp.: Update on Taxonomy, Clinical Aspects, and Emerging Antimicrobial Resistance. Clin Microbiol Rev. 2019 Sep 18;32(4). doi:10.1128/CMR.00002-19

6. Schaffer JN, Pearson MM. *Proteus mirabilis* and Urinary Tract Infections. Microbiol Spectr. 2015 Sep 4;3(5). doi:10.1128/microbiolspec.UTI-0017-2013

7. Loughran AJ, Orihuela CJ, Tuomanen EI. *Streptococcus pneumoniae* : Invasion and Inflammation. Microbiol Spectr. 2019 Apr 12;7(2). doi:10.1128/microbiolspec.GPP3-0004-2018

8. Feasey NA, Dougan G, Kingsley RA, Heyderman RS, Gordon MA. Invasive non-typhoidal salmonella disease: an emerging and neglected tropical disease in Africa. The Lancet. 2012 Jun;379(9835):2489–99. doi:10.1016/S0140-6736(11)61752-2

9. Tong SYC, Davis JS, Eichenberger E, Holland TL, Fowler VG. Staphylococcus aureus Infections: Epidemiology, Pathophysiology, Clinical Manifestations, and Management. Clin Microbiol Rev. 2015 Jul;28(3):603–61. doi:10.1128/CMR.00134-14

10. Bottone EJ. *Bacillus cereus* , a Volatile Human Pathogen. Clin Microbiol Rev. 2010 Apr;23(2):382–98. doi:10.1128/CMR.00073-09

11. Paczosa MK, Mecsas J. Klebsiella pneumoniae: Going on the Offense with a Strong Defense. Microbiology and Molecular Biology Reviews. 2016 Sep;80(3):629–61. doi:10.1128/MMBR.00078-15

12. Qin S, Xiao W, Zhou C, Pu Q, Deng X, Lan L, et al. Pseudomonas aeruginosa: pathogenesis, virulence factors, antibiotic resistance, interaction with host, technology advances and emerging therapeutics. Signal Transduct Target Ther. 2022 Jun 25;7(1):199. doi:10.1038/s41392-022-01056-1

13. Arias CA, Murray BE. The rise of the Enterococcus: beyond vancomycin resistance. Nat Rev Microbiol. 2012 Apr 16;10(4):266–78. doi:10.1038/nrmicro2761

14. Peleg AY, Seifert H, Paterson DL. *Acinetobacter baumannii* : Emergence of a Successful Pathogen. Clin Microbiol Rev. 2008 Jul;21(3):538–82. doi:10.1128/CMR.00058-07

15. Brooke JS. Stenotrophomonas maltophilia: an Emerging Global Opportunistic Pathogen. Clin Microbiol Rev. 2012 Jan;25(1):2–41. doi:10.1128/CMR.00019-11

16. Byrd AL, Belkaid Y, Segre JA. The human skin microbiome. Nat Rev Microbiol. 2018 Mar 15;16(3):143–55. doi:10.1038/nrmicro.2017.157

17. Fierer N, Leung PM, Lappan R, Eisenhofer R, Ricci F, Holland SI, et al. Guidelines for preventing and reporting contamination in low-biomass microbiome studies. Nat Microbiol. 2025 Jun 20;10(7):1570–80. doi:10.1038/s41564-025-02035-2

18. Eisenhofer R, Minich JJ, Marotz C, Cooper A, Knight R, Weyrich LS. Contamination in Low Microbial Biomass Microbiome Studies: Issues and Recommendations. Trends Microbiol. 2019 Feb;27(2):105–17. doi:10.1016/j.tim.2018.11.003

19. Salter SJ, Cox MJ, Turek EM, Calus ST, Cookson WO, Moffatt MF, et al. Reagent and laboratory contamination can critically impact sequence-based microbiome analyses. BMC Biol. 2014 Dec 12;12(1):87. doi:10.1186/s12915-014-0087-z

20. Marriott D, Stark D, Harkness J. *Veillonella parvula* Discitis and Secondary Bacteremia: a Rare Infection Complicating Endoscopy and Colonoscopy? J Clin Microbiol. 2007 Feb;45(2):672–4. doi:10.1128/JCM.01633-06
